# Vaccine Hesitancy, Knowledge, and COVID-19 Vaccination in a Sample of Italian and Albanian Healthcare Students Attending an University in Albania

**DOI:** 10.3390/tropicalmed9030057

**Published:** 2024-02-29

**Authors:** Ersilia Buonomo, Fabian Cenko, Gaia Piunno, Daniele Di Giovanni, Enkeleda Gjini, Bora Kërpi, Mariachiara Carestia, Stefania Moramarco, Cristiana Ferrari, Luca Coppeta

**Affiliations:** 1 Department of Biomedicine and Prevention, University of Rome “Tor Vergata”, 00133 Rome, Italy; buonome@uniroma2.it (E.B.); gaia.piunno@students.uniroma2.eu (G.P.);; 2 Faculty of Medicine, Catholic University of “Our Lady of Good Counsel”, 1000 Tirane, Albania; f.cenko@unizkm.al (F.C.); e.gjini@unizkm.al (E.G.); b.kerpi@unizkm.al (B.K.); luca.coppeta@ptvonline.it (L.C.); 3 Industrial Engineering Department, University of Rome “Tor Vergata”, 00133 Rome, Italy; 4 Faculty of Medicine, Unicamillus, Saint Camillus International University of Health Sciences, 00131 Rome, Italy; 5 Department of Occupational Medicine, University of Rome “Tor Vergata”, 00133 Rome, Italy; cristiana.ferrari@ptvonline.it

**Keywords:** vaccine hesitancy, vaccine knowledge, COVID-19 vaccination, medical sciences students, Albania

## Abstract

Background: Vaccine hesitancy (VH) has increased over the past decade with large geographical variations between countries, posing a threat to global public health. This phenomenon is growing in the general population as well as among healthcare workers (HCWs), who are the most reliable source of vaccine-related information for patients. Special attention must therefore be paid to medical students, who are the future HCWs. Methods: We conducted a cross-sectional study (November 2022–January 2023) on all the Albanian and Italian students attending medical science courses at the Catholic University “Our Lady of Good Counsel” (Tirane, Albania) to investigate VH and the factors contributing to it (using the Vaccination Attitude Examination Scale-VAX), including COVID-19 vaccination. Vaccine knowledge was assessed using the Zingg and Siegrist Scale. Students were asked to voluntarily answer an anonymous questionnaire. Results: 689 questionnaires were collected (58.8% Albanians, 72.3% female; 70.4% aged 20–25 years; 70.4% attending the Medicine and Surgery course). Generally, students showed low VH, especially Italians (*p* < 0.001); however, some hesitancy was observed regarding the potential long-term effects of vaccines, especially among Albanians (*p* < 0.05). The results also showed a significant difference in vaccine knowledge scores between different course years (χ^2^  =  90.058; df  =  40; *p* = < 0.001) and different degree courses (χ^2^  =  89.932; df = 40; *p*  = < 0.001). With regard to COVID-19 vaccination, being of Albanian origin significantly increases the risk of not being vaccinated (OR = 7.215; 95%CI 3.816–13.640, *p* < 0.001), highlighting possible differences in vaccine coverage and policy between the two countries. Conclusion: Vaccine hesitancy should be addressed at early stages during medical sciences courses, in order to protect future healthcare workers, to preserve essential health services, and reduce the risk of further pandemics.

## 1. Introduction 

Vaccinations are widely acknowledged as safe, effective, and one of the most successful preventive measures [1], estimated to prevent infection in 4–5 million people worldwide annually prior to the COVID-19 pandemic [2]. However, global vaccine confidence has diminished among the general public since 2020, partly due to the widespread dissemination of vaccine-related concerns and controversies in the media, leading to a substantial public perception that vaccines may pose risks [3]. This decline has contributed to Vaccine Hesitancy (VH), defined as a “delay in acceptance or refusal of vaccination despite the availability of vaccination services” [4]. According to the World Health Organization (WHO), vaccine hesitancy has risen over the last decade, becoming one of the ten threats to global health in 2019 [5]. The latest European Commission report highlights significant geographic variation in vaccine confidence, with many Eastern and Central European countries experiencing a worrisome decrease [2]. Notably, the Western Balkans face vaccine hesitancy challenges [6], yet there are limited data on the phenomenon and its distribution. Regarding the recent COVID-19 vaccination campaign, a study in Europe revealed considerable heterogeneity, with lower hesitancy rates in countries like Italy (below 20%) compared to higher rates in Balkan countries like Bulgaria (over 60%), especially among women [7]. 

As of now, for some other countries, such as Albania, still-limited research has explored the occurrence of VH [8,9,10]. Despite experiencing economic growth, the implementation of universal health insurance, increased spending on health services, and the provision of free childhood immunization, Albania is confronting a decline in childhood immunization coverage [11]. 

Understanding vaccine hesitancy involves recognizing the intricate decision-making process influenced by cognitive, emotional, social, and spiritual factors. Moreover, historical, political, and socio-cultural contexts shape vaccination attitudes [12,13]. VH is a complex challenge, varying across time, place, and socio-demographics. Broader factors contributing to hesitancy, such as public health policies, vaccine policies, communication strategies, media influence, and healthcare professionals’ roles, should be considered [14].

The report published by the European Commission declared that vaccine confidence among European healthcare staff remained high in 2022, with large differences among countries, and low rate of vaccine confidence found in Eastern countries such as Croatia and Slovakia (nearly 60%) compared to Mediterranean countries, such as Italy and Spain (between 85 and 90%) [2]. 

Healthcare workers (HCWs) are widely regarded as the most reliable source of vaccine-related information for patients [15]. They are uniquely positioned to grasp the concerns of hesitant patients, address their anxieties, and articulate the advantages of vaccination. Studies on HCWs have primarily focused on nurses, most of whom exhibit confidence in vaccines and favor childhood immunizations. However, upon closer examination of their beliefs, a notable proportion expressed vaccine hesitancy [8,16]. As a matter of fact, recent studies indicate a growing phenomenon of vaccine hesitancy among HCWs themselves [17], spanning considerations for personal vaccination, that of their children, or even for their patients [18]. Special attention must then be paid to a subclass of HCWs, the medical students, who are future health professionals. Medical students, compared to their counterparts, tend to demonstrate greater acceptance of vaccines, although their perception of vaccination as a crucial anti-epidemic measure may vary. Notably, students, in contrast to other healthcare workers, exhibit a more altruistic inclination, prioritizing patient benefits over their own [19]. Despite this, global inadequacy in knowledge regarding occupational immunizations is prevalent among healthcare students, with a discernible correlation to their year of study. First-year medical or nursing school students often exhibit limited awareness of vaccines and the diseases they can prevent, underscoring the need for improvement in both basic and continuing education on vaccines [20]. When considering Albania, there is a lack of information about students’ attitudes toward vaccination. When comparing the determinants of voluntary and mandatory vaccination among Albanian university students, vaccine safety and efficacy were found to be hindering factors in vaccination [21]. It is therefore necessary to study Albania’s state of the art in matters of VH, as the country’s situation in terms of vaccines is rather delicate. Identifying the determinants of vaccine hesitancy and the attitudes of medical students toward vaccines in this context is therefore crucial to understanding what interventions may be most effective in promoting vaccination. The present study aimed to investigate VH among students attending medical science courses at a University in Albania and the factors contributing to it, also focusing on COVID-19 and vaccination predictors. 

## 2. Materials and Methods

### 2.1. Study Design and Sample

Between November 2022 and January 2023, we conducted an observational cross-sectional study consisting of data collected through a structured survey offered to students of the Catholic University “Our Lady of Good Counsel” in Tirane (Albania). It should be pointed out that it is a mixed university hosting both Europeans and non-Europeans, since it has an academic and scientific agreement with the Italian University of Rome “Tor Vergata”. Participation in the study was on a voluntary basis, so the only requirement was enrolment in any healthcare academic program provided by the Faculties of Medicine and Surgery of “Our Lady of Good Counsel” University, i.e., Medicine and Surgery, and other medical science courses, i.e., Nursing, Pharmacy, Physiotherapy, and Dentistry. All the respondents were asked to answer a self-administered anonymous printed questionnaire, available in both the Italian and Albanian language. To participate to the survey, participants had to provide informed consent. Students were informed that their right to withdraw had no impact on their academic standing or grades. There were no incentives or rewards offered for taking part in the study. The study was approved by the Ethics Committee of the University of Rome “Tor Vergata” (Rome) (n.210/22, 2022).

### 2.2. Questionnaire

Following a comprehensive review of prior studies and the relevant literature, we developed a semi-structured questionnaire comprising distinct sections. 

The first section encompassed inquiries targeting socio-demographic information, vaccinal status, and vaccine-related health conditions of the participants, including personal vaccination history, COVID-19 experiences, and health behaviors related to vaccination.

In the second section, the validated Italian version of the Vaccination Attitude Examination Scale (VAX) was presented [22], adapted to the needs of the study. The VAX scale is used to measure vaccination attitudes and vaccine hesitancy in HCWs; a positive attitude is indicated by the lowest scores, while the highest score indicates VH. The scale investigates the general propensity to vaccination through four domains: mistrust of vaccine benefits, worries about unforeseen future effects, concerns about commercial profiteering, and preference for natural immunity. To better align with the study‘s objectives, an additional question was incorporated, to assess students’ perceptions of their future roles as healthcare professionals, a supplementary question: “I consider it my duty as a health professional to educate patients about vaccinations”. The 12 questions were individually scored on a Likert scale ranging from 1 to 5, where 1 denoted “Totally disagree”, and 5 denoted “Totally agree”. To accurately represent this factor, four scale items were reversed, and, consequently, the score was inverted during the presentation of findings.

The third section featured the Zingg and Siegrist Scale [23], adapted to the study‘s requirements. This previously validated scale comprised 10 questions with response options of “yes”, “no”, or “I do not know”, designed to delve into respondents‘ knowledge of vaccines. Regarding the evaluation of general knowledge about vaccines, data analysis involved creating a variable based on the sum of scores obtained in the test, with correct answers receiving 1 point, and incorrect or “I do not know” responses receiving 0 points. 

The questionnaires were submitted in the classrooms by two bilingual coordinators, to support any possible challenge in comprehension.

### 2.3. Sample Size 

We calculated the sample size considering a margin of error of plus/minus 5 percentage points, with 95% confidence, and setting a standard deviation of 0.5, which produces a conservative estimate of the variance. At least 331 subjects were required for the analysis. To obtain a confidence of 99%, 521 subjects were required for the analysis.

### 2.4. Statistical Analysis

Socio-demographic data and health status information derived from the initial section of the semi-structured questionnaire were reported as a descriptive analysis (numbers and percentages), as a total then split by nationality.

When assessing vaccination hesitancy through the VAX scale, Cronbach‘s alpha was employed to determine the internal consistency reliability of the measurement items, with an alpha value of 0.7 or higher indicative of acceptable reliability. Results were presented as mean values ± standard deviations (SD). T-tests for independent variables were used to compare differences in mean values of VAX scores between Albanian and Italian students.

The association between socio-demographic data and the respondents’ general knowledge about vaccines was assessed using a chi-square test. Simple and multiple predictor logistic regression analyses were performed with COVID-19 vaccination status as the outcome. 

A binary logistic regression was conducted to identify predictors of COVID-19 vaccination. Dichotomization was applied to the religion variable, by categorizing those practicing religion and those identifying as atheists/agnostics. To evaluate knowledge, a dichotomized value was assigned to the total questionnaire score, categorized as either 0 to 5 (indicating low knowledge) or 6 to 10 (indicating high knowledge). Because there were multiple independent variables, a stepwise forward regression approach was used. A *p*-value < 0.05 was considered statistically significant for all analyses. IBM SPSS Statistics (version 25) was utilized for the statistical elaboration of the data.

## 3. Results

A total of 689 questionnaires were filled in by students. The interview covered 38.5% of all the students enrolled in the Faculty of Medicine and Pharmacy. Among the participants, 498 (72.3%) were female, and 191 (27.7%) were male. The majority (70.4%) fell within the 20- to 25-year-old age range. Overall, 485 (70.4%) participants were enrolled in the Medicine and Surgery course, while the remaining sample comprised students from other medical science degree courses such as Nursing, Pharmacy, Physiotherapy, and Dentistry. Concerning religion, 484 students declared practicing a religious faith (41.7% were Catholic Christians, 24.5% were Muslims, and 4.1% were Catholic Orthodox), while the remaining 205 declared themselves atheist or agnostic (29.8%). The participants‘ nationality was 58.8% (408) Albanian and 40.6% (281) Italian. None of the questionnaires had missing information, so none were excluded. Table 1 illustrates the principal socio-demographic characteristics of the study group.

Table 2 presents the vaccinal status and vaccine-related health behavior. Of the sample, 82% (565) declared being vaccinated against COVID-19, while only 16.2% (112) had received a flu vaccine the previous year. Exploring the sample‘s vaccination background, 11.5% (79) of the students had refused a recommended vaccine at least once, and 13.9% (96) had delayed at least once a vaccination for reasons other than allergy or illness.

Regarding the adapted VAX scale for assessing VH among healthcare students, Table 3 provides detailed descriptive statistics for each item. In analyzing responses to the first domain, “Mistrust of vaccine benefit”, an overall agreement with positive statements was observed, indicating confidence in vaccination. The reversed score for the item “I can rely on vaccines to stop serious infectious diseases” also suggests a high degree of trust in vaccines (1.87 ± 1.00). 

Concerning “Worries about unforeseen future effects”, the sample expressed some doubts, particularly in the item “Although most vaccines appear to be safe, there may be problems that we have not yet discovered”, with a mean score of 3.59 ± 0.98. Responses to the “Concerns about commercial profiteering” factor tended to disagree with the proposed statements, especially for the item “Vaccination programs are a big con” (1.91 ± 1.07), indicating a general lack of belief in conspiracies behind vaccination programs. Statements about “Preference for natural immunity” underline some hesitation toward immunity conferred by vaccines, with the agreement that “Natural exposure to viruses and germs gives the safest protection” (2.72 ± 1.14).

When asked about their duty to educate patients about vaccination as future health professionals, the sample agreed with this statement (1.94 ± 0.98). When splitting the sample by nationality, Italian students had slightly lower mean score responses than their Albanian counterparts for almost all items, indicating greater vaccination hesitancy among the latter. The overall internal consistency was 0.848, and Cronbach’s alpha values ranged from 0.80 to 0.90, suggesting each item contributed to the overall reliability of the scale [24]. 

**Figure 1:**
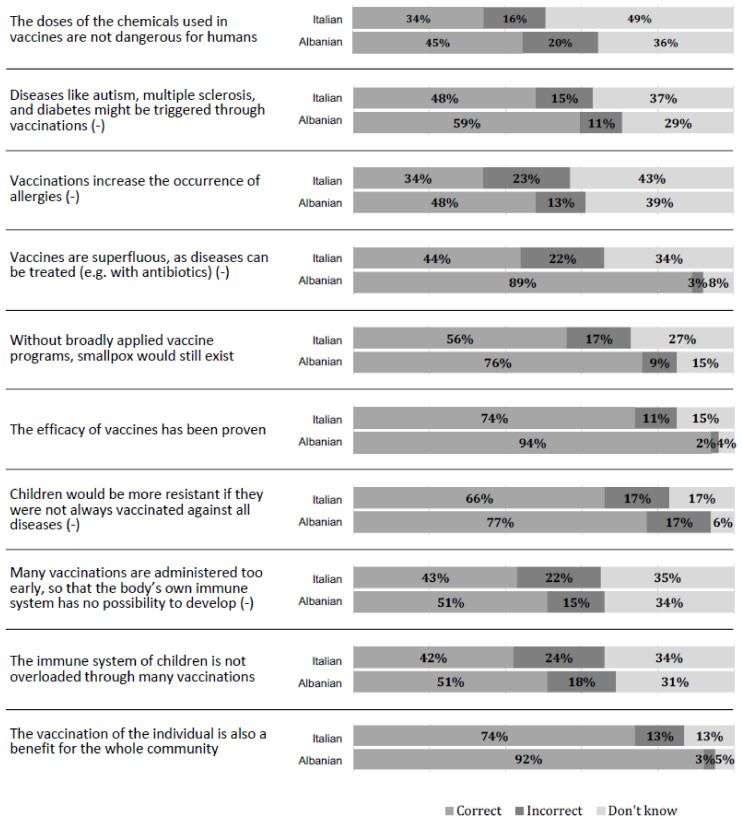
Knowledge item response distributions, divided by nationality (*n* = 689). Items having an incorrect statement were indicated by (−).

Concerning the assessment of general knowledge of vaccines (Figure 1), the mean score of the sample was 5.51 ± 2.66. Only 36 students (5.2%) answered all ten questions correctly. Knowledge was significantly higher (*p* < 0.001) among Italian respondents (6.65 ± 2.34) than among Albanians (4.72 ± 2.58). A significant difference was observed in the overall knowledge of vaccines score among different course years (χ^2^ =  90.058; df  =  40; *p*  = < 0.001) and different degree courses (χ^2^  =  89.932; df  =  40; *p*  = < 0.001), indicating that students attending higher years of courses and the Medicine and Surgery course, regardless of nationality, were significantly more informed about vaccination than students attending lower years of courses and other medical science courses.

The logistic regression (Table 4), performed to identify the main statistically significant predictors of being vaccinated against COVID-19, showed that Albanian students had a higher risk of not being vaccinated (OR = 7.215; 95%CI 3.816–13.640, *p* < 0.001). Conversely, low knowledge about vaccines was inversely associated with the likelihood of not having received the vaccine (OR = 2.378; 95%CI 1.513–3.739, *p* < 0.001), indicating that those with greater knowledge were more likely to be vaccinated. 

## 4. Discussion

The current survey study involved future health professionals, offering important information regarding the variables surrounding Vaccine Hesitancy among medical sciences students. According to our findings, students enrolled in medical science courses in Albania, both Italians and Albanians, exhibited a considerable level of confidence in vaccines and public health organizations. However, some hesitancy was evident concerning the unforeseen future effects of vaccines. Notably, the lowest mean scores were reported in the reversed domain “Mistrust of vaccine benefits”, with statistically significant differences between Italian and Albanian students, aligning with the results of the study conducted by Tomietto et al. on Italian nursing professionals [22]. Specifically, from our findings, participants expressed the highest level of agreement with the item “I feel safe after being vaccinated” (mean value 2.1 vs. 2.5). Conversely, the highest scores were observed in the domain “Worries about unforeseen future effects”, particularly when students, both Italians and Albanians, responded to statements “Although most vaccines appear to be safe, there may be problems that we have not yet discovered” (mean 2.5 vs. 2.6; although not statistically significant) and “I worry about the unknown effects of vaccines in the future” (mean 2.9 vs. 3.4).

Confidence in vaccine efficacy was found to be the best predictor of vaccine acceptance among healthcare operators in previously published studies, and interventions aimed at improving trust in vaccine effectiveness can help to achieve higher rate of vaccine acceptance among those operators [17]. Safety concerns on vaccines are related to higher vaccine hesitation even if vaccine hesitancy should not be equated to vaccine safety, that is only one determinant of the hesitation [4,25].

To assess their role as future healthcare professionals in promoting vaccine campaigns, students answered the item “I consider it my duty as a health professional to educate patients about vaccinations”, showing a high inclination to perceive it as a key responsibility during immunization programs. However, a significant difference was reported between the two nationalities (mean value 1 vs. 2.1). Therefore, it is crucial to emphasize the importance of providing proper education and technical skills related to vaccines and vaccination for the upcoming generation of healthcare professionals, especially for Albanian students, to enhance vaccine confidence even among their future patients, since low vaccine confidence in healthcare staff has been recognized as an independent predictor of parental VH [8].

Our analysis revealed that older medical students attending the latest years of courses showed greater confidence in vaccination and possessed more knowledge about it. This finding aligns with the European Union report on vaccine confidence [2], which indicated that younger age groups and lower levels of education tend to have lower confidence compared to their counterparts. 

Concerning COVID-19 vaccination, being Albanian significantly increases the risk of not being vaccinated for COVID-19, highlighting differences in health legislation across the two countries: the lower vaccine confidence among Albanian students should be contextualized within the cultural, geographical, and public health policy frameworks [9,26,27]. On the other hand, individuals with low knowledge about vaccines were less likely to have received the COVID-19 vaccine, emphasizing the importance of educational initiatives in promoting vaccination [28]. It is also noteworthy that students attending the Medicine and Surgery course were significantly more informed about vaccinations than their peers. Further investigation will be necessary to clarify whether this result may be attributed to encouragement by the academic staff, as reported by Kongo et al. [21].

### 4.1. Implications for Future

It is necessary to implement government strategies to promote vaccinations by highlighting the efficacy and safety of the vaccine. This can be achieved by emphasizing the lower incidence rate of the disease and complications among the vaccinated compared to others [17]. Moreover, recommendations on vaccination by occupational physicians can significantly influence the attitudes of healthcare workers. Studies suggest that the educational contribution of occupational physicians can play a crucial role, particularly for nurses, who are among the most hesitant groups [29]. Therefore, it is essential for occupational physicians to conduct educational and promotive vaccination campaigns [30].

### 4.2. Limitations 

One possible limitation of our study is that participation in the survey was not mandatory for students, which may have resulted in sample selection bias and limited the generalizability of the results. However, we registered only one refusal and no withdraw. It is possible that subjects who were against vaccination were more reluctant to respond than those who wanted to be vaccinated. 

Not having performed a pilot survey to assess the validity and reliability of the instruments might be considered a limitation of the study, despite the main questionnaires used being validated in the scientific literature.

The questionnaires were administered in classrooms to students attending the lessons; therefore, the low coverage registered for some courses could have been due to students attending practical training. 

Additionally, the study lacks details on COVID-19 immunization, such as the type of vaccine, if fully or partially immunized, and the booster dose. Furthermore, other relevant information could have been left out, such as the low percentage of students being vaccinated against influenza during the previous year; this condition was not explored since it was beyond the aim of the study. 

## 5. Conclusion

Research on vaccine hesitancy among medical science students remains underrepresented in the literature, particularly in Eastern European countries such as Albania. This study highlights that medical sciences students have trust in vaccines and gain vaccine knowledge throughout the academic training course, all conditions essential to promote positive immunization attitudes among their future patients. Nevertheless, widespread and interdisciplinary full-package training on vaccine safety and efficacy should be a priority of medical universities to address at an early stage the vaccine-related concerns and reluctance held by hesitant future healthcare professionals.

## Figures and Tables

**Table 1:** Demographic features of the study population, divided by nationality.

		Italian *n* = 281	%	Albanian *n* = 408	%	Total *n* = 689	% of Sample
**Gender**	F	190	67.6	308	75.5	498	72.3
	M	91	32.4	100	24.5	191	27.7
**Age**	<20	37	13.2	34	8.3	71	10.3
	20–25	192	68.3	293	71.8	485	70.4
	26–34	39	13.9	56	13.7	95	13.8
	>35	13	4.6	25	6.2	38	5.5
**Religion**	Religious	223	79.4	261	64.0	484	70.3
	Atheist/Agnostic	58	20.6	147	36.0	205	29.7
**Degree Course**	Physiotherapy	56	19.9	22	5.4	78	11.3
	Nursing	9	3.2	35	8.6	44	6.4
	Medicine and Surgery	187	66.5	298	73.0	485	70.4
	Dentistry	9	3.2	10	2.5	19	2.8
	Pharmacy	20	7.2	43	10.5	63	9.1

**Table 2:** Description of the vaccinal status and vaccine-related health conditions of the sample, divided by nationality (*n* = 689).

Question or Statement		Italian *n* = 281	%	Albanian *n* = 408	%	Total *n* = 689	% of Sample
**I did at least one COVID-19 swab**	No	5	1.8	104	25.5	109	15.8
	Yes	276	98.2	304	74.5	580	84.2
**I have contracted COVID-19** **disease at least once**	No	102	36.3	140	34.4	242	35.1
	Yes	179	63.7	268	65.7	447	64.9
**I took care of a person with COVID-19**	No	178	63.3	257	63.0	435	63.5
	Yes	103	36.7	151	37.0	254	36.8
**A family member of mine** **contracted COVID-19 disease**	No	26	9.3	94	23.0	120	17.4
	Yes	255	90.7	314	77.0	569	82.6
**I had the COVID-19 vaccine**	No	12	4.3	112	27.5	124	18.0
	Yes	269	95.7	296	72.5	565	82.0
**I postponed at least one other vaccination because of the COVID-19 vaccination**	No	267	95.0	373	91.4	640	92.9
	Yes	14	5.0	35	8.6	49	7.1
**I had my HepB vaccine booster**	No	177	63.0	344	84.3	521	75.6
	Yes	104	36.0	64	15.7	168	24.4
**I had the flu vaccine last year**	No	218	77.6	359	88.0	577	83.7
	Yes	63	22.4	49	12.0	112	16.2
**I have refused a recommended vaccine** **at least once**	No	262	93.2	348	85.3	610	88.5
	Yes	19	6.8	60	14.7	79	11.5
**I have delayed a vaccination at least once for reasons other than allergy or illness**	No	250	89.0	343	84.1	593	86.1
	Yes	31	11.0	65	15.9	96	13.9

**Table 3:** Descriptive statistics of the vaccination attitudes, opinions, and confidence about vaccines of the sample. (R) denotes reversed items.

Factor	Item	Mean ±SD	Italians Mean ±SD	Albanians Mean ±SD	Two-Tailed Significance
**Mistrust of vaccine benefit**	I feel safe after being vaccinated (R)	2.37 ± 1.08	2.12 ± 0.99	2.55 ± 1.11	0.000
	I can rely on vaccines to stop serious infectious diseases (R)	1.87 ± 1.00	1.49 ± 0.69	2.15 ± 1.09	0.000
	I feel protected after getting vaccinated (R)	2.27 ± 1.05	2.05 ± 0.96	2.42 ± 1.09	0.000
**Worries about** **unforeseen future effects**	Although most vaccines appear to be safe, there may be problems that we have not yet discovered	3.59 ± 0.98	3.57 ± 0.94	3.61 ± 1.01	0.560
	Vaccines can cause unforeseen problems in children	2.99 ± 1.09	2.89 ± 1.05	3.06 ± 1.13	0.045
	I worry about the unknown effects of vaccines in the future	3.20 ± 1.13	2.91 ± 1.18	3.41 ± 1.06	0.000
**Concerns about commercial** **profiteering**	Vaccines make a lot of money for pharmaceutical companies, but do not do much for regular people	2.38 ± 1.10	1.98 ± 1.00	2.67 ± 1.09	0.000
	Authorities promote vaccination for financial gain, not for people’s health	2.22 ± 1.09	1.84 ± 0.93	2.49 ± 1.12	0.000
	Vaccination programs are a big con	1.91 ± 1.07	1.57 ± 0.87	2.15 ± 1.13	0.000
**Preference for natural immunity**	Natural immunity lasts longer than a vaccination	3.03 ± 1.19	2.55 ± 1.11	3.37 ± 1.12	0.000
	Natural exposure to viruses and germs gives the safest protection	2.72 ± 1.14	2.28 ± 1.02	3.04 ± 1.12	0.000
	I consider it my duty as a health professional to educate patients about vaccinations (R)	1.94 ± 0.98	1.06 ± 0.86	2.18 ± 0.99	0.000

**Table 4:** Potential determinants of no COVID-19 vaccine uptake in medical students (binary logistic regression).

							95% C.I. for EXP(B)	
	B	Standard Error	Wald	Df	Significance	Exp(B)	Lower	Upper
**Female Gender**	0.229	0.238	0.926	1	0.336	1.257	0.789	2.003
**Low Vaccine Knowledge**	0.866	0.231	14.089	1	0.000	2.378	1.513	3.739
**Albanian Nationality**	0.976	0.325	36.988	1	0.000	7.215	3.816	13.640
**Traditional Religion**	−0.240	0.231	1.079	1	0.299	0.787	0.501	1.237
**Constant**	0.668	0.268	6.219	1	0.013	1.950		

## Data Availability

Data are available on request.
